# Identification of proteins involved in neural progenitor cell targeting of gliomas

**DOI:** 10.1186/1471-2407-9-206

**Published:** 2009-06-26

**Authors:** Karin Staflin, Thole Zuchner, Gabriella Honeth, Anna Darabi, Cecilia Lundberg

**Affiliations:** 1 CNS Gene Therapy Unit, Dept Experimental Medical Science, Lund University, Lund, Sweden; 2 Ultrasensitive Protein Detection Unit, Institute of Bioanalytical Chemistry, Leipzig University, Leipzig, Germany; 3 Dept Oncology, Clinical Sciences, Lund University, Lund, Sweden; 4 Dept Molecular and Experimental Medicine, The Scripps Research Institute, La Jolla, CA, USA; 5 Glioma Immunotherapy Unit, The Rausing Laboratory, Lund University, Lund, Sweden

## Abstract

**Background:**

Glioblastoma are highly aggressive tumors with an average survival time of 12 months with currently available treatment. We have previously shown that specific embryonic neural progenitor cells (NPC) have the potential to target glioma growth in the CNS of rats. The neural progenitor cell treatment can cure approximately 40% of the animals with malignant gliomas with no trace of a tumor burden 6 months after finishing the experiment. Furthermore, the NPCs have been shown to respond to signals from the tumor environment resulting in specific migration towards the tumor. Based on these results we wanted to investigate what factors could influence the growth and progression of gliomas in our rodent model.

**Methods:**

Using microarrays we screened for candidate genes involved in the functional mechanism of tumor inhibition by comparing glioma cell lines to neural progenitor cells with or without anti-tumor activity. The expression of candidate genes was confirmed at RNA level by quantitative RT-PCR and at the protein level by Western blots and immunocytochemistry. Moreover, we have developed *in vitro *assays to mimic the antitumor effect seen *in vivo*.

**Results:**

We identified several targets involved in glioma growth and migration, specifically CXCL1, CD81, TPT1, Gas6 and AXL proteins. We further showed that follistatin secretion from the NPC has the potential to decrease tumor proliferation. *In vitro *co-cultures of NPC and tumor cells resulted in the inhibition of tumor growth. The addition of antibodies against proteins selected by gene and protein expression analysis either increased or decreased the proliferation rate of the glioma cell lines *in vitro*.

**Conclusion:**

These results suggest that these identified factors might be useful starting points for performing future experiments directed towards a potential therapy against malignant gliomas.

## Background

Current treatment for GBM has proven ineffective mainly due to the disseminating nature of the tumor in addition to the resistance to irradiation and chemotherapy. Novel approaches have tried to utilize the migratory potential of neural progenitor cells (NPC) and their ability to functionally integrate into host tissues to target this invasive tumor [1-6]. In previous studies we have shown that specific embryonic rat neural progenitor cells have the ability to target malignant gliomas in the rat striatum [7]. These cells can inhibit the growth of a solid tumor in the animals, preventing proliferation as well as invasion. The embryonic neural progenitor cells are able to cure up to 40% of the animals in co-inoculation experiments, with no trace of a tumor burden 6 months after finishing the experiment. This significant antitumor effect is quite remarkable in light of the poor prognosis displayed by glioma patients undergoing current treatment regimens. We have further shown that these NPC upon transplantation at a distance from a three day pre-established primary tumor can migrate to the tumor and elicit an antitumor effect [8]. This antitumor effect is demonstrated by a significant reduction in tumor volume that is evident at early time points and can be directly translated into survival studies of three day pre-established tumors, as shown by prolonged survival times for treated animals [8]. The objective of this study was to find candidate genes involved in influencing tumor growth based on the strong antitumor effect shown by the NPC. We hereby wanted to identify potential targets or factors, which could prove effective in treating malignant gliomas without the use of cell based therapy. This was done by comparing gene and protein levels of NPCs with antitumor effects vs. NPCs without antitumor effects as well as comparing gene and protein levels of sensitive versus insensitive tumors. A functional cross-comparison revealed interesting gene and protein candidates that are both involved in tumor growth as well as in tumor growth suppression. These candidates were analyzed in initial functional assays.

## Methods

### Cell lines

The rat glioma cell lines, N25, N29 and N32 (syngeneic with the Fisher 344 line) were established and maintained as previously described [9,10] in RPMI (GIBCO) supplemented with 10% fetal bovine serum (FBS, Sigma). The rat embryonic neural progenitor cell lines HiB5 from hippocampus [11], ST14A from striatum [12] and RN33B derived from medullary raphe [13] were conditionally immortalized with the temperature sensitive SV40 large T and propagated as previously described [7,8] at the permissive temperature of 33°C. For RNA and histological preparations, the cells were cultured under the non-permissive temperature of 39°C in serum free medium for three days before harvesting or staining.

### Microarray design

cDNA/oligo microarray slides were obtained from the Swegene DNA Microarray Resource Center at Lund University, Sweden http://swegene.onk.lu.se. RNA, cDNA synthesis, labeling, quality control and hybridizations were performed according to previous publications [14]. The labeled cDNA was hybridized to rat 18 k microarray slides which represents 5720 genes and scanned in a G2565AA Agilent DNA Microarray Scanner (Agilent Technologies, Palo Alto, CA, USA). Spot analysis was performed using GenePix 4.0 software (GenePix, Foster City, CA, USA) and the subsequent data matrix was uploaded to the BioArray Software Environment (BASE) [15]. Normalizations, correction for bad quality spots, filter setups, hierarchical clustering, and analysis was done in BASE according to previous publication [16]. Results were analyzed with the software package BASE 1.2.17 (available at http://base.onk.lu.se).

To analyze the genomic data, the following analysis was performed: (I) cluster analysis of overall expression profiles in order to find relations between cell lines; (II) statistical analysis and (III) literature research and use of gene ontology databases to distinguish between relevant and non-relevant genes. Hierarchical clustering was performed on 4,582 reporters remaining after data pre-processing and filtering using a bottom-up approach with center of mass linkage and Pearson correlation distance.

Statistical analysis required filtering of the dataset according to the classification of the signals measured in 'absent' (A) or 'present' (P). For statistical reasons, genes that were not present in the entire microarray analyses were excluded from the related comparisons [17]. Each cell line was analyzed on a separate microarray and results were compared afterwards.

The standard deviation in percent was calculated for the different groups and only genes below a percent standard deviation of 0.2 were taken into account. The minimum fold change value was set to 2. Resulting genes were classified according to their functions against the background of gene-ontology databases and the underlying literature and experimental background. Statistical analysis was performed using the software SAM (available at http://www-stat.stanford.edu/~tibs/SAM/), which allows the detection of falsely positive genes (family-wise error rate) by averaging the amount of genes classified as significant over chosen permutations of data between the groups. The false discovery rate was set to false discovery rate ≤ 1·10^-4^. After statistical analysis, genes were selected according to the highest fold change differences and their probable involvement in cell proliferation.

### Real-time quantitative RT-PCR

Real-time quantitative RT-PCR was carried out on cDNA from the glioma cell lines and the neural progenitor cell lines using SYBR Green dye supermix for the ABI system (Invitrogen, Carlsbad, Ca). Briefly, total RNA was prepared from 80% confluent T75 culture flasks, by using TRIzol (Invitrogen, Carlsbad, Ca) according to manufacturers protocol. In case of the progenitor cells, the cells were grown in serum-free conditions and at the permissive temperature of 37°C for 3 days. To remove remaining genomic DNA, the samples were purified again and DNase I treated by using RNeasy (Qiagen, Valencia, CA, USA). cDNA preparations were conducted, using 1.5 μg of RNA, according to manufacturers protocol (Superscript III reverse transcriptase; Invitrogen) with the addition of RNaseI (Promega). SYBR green PCR was done using a 7900HT Fast Real-Time System (Applied Biosystems, Foster City, CA). The PCR reactions were setup in 20 μl volumes using a 2× SYBR Green PCR master mix (Invitrogen, Carsbad, CA), 900 μM of each primer. In addition negative internal controls were used (no reverse transcriptase) and GAPDH was used as a reference gene. The following amplification program was used: 50°C for 2 minutes, 95°C for 10 minutes, 40 cycles of 95°C for 15 seconds, 60°C for 1 minute and 74°C 30 seconds and finally an additional 15 seconds of 95°C. After cycling, relative quantification of the specific mRNA expressions in the different cells was calculated and compared. Relative DNA levels were quantified using the comparative Ct-method according to User Bulletin #2, supplied by Applied Biosystems http://www.appliedbiosystems.com. The following primers where used (forward and reverse): Gas 6: 5'CTCCTACTCCTGCCTCT'3, 5'GCTGCCTGGGGAGTTGA'3, CD81: 5' CATCCAGGAGTCCCAGT'3, 5'CAGCGTATTGGAGCCACA'3, AXL: 5'CTCTGGCTTCAAGATGCTGT'3, 5'GTGGTGACTCCCTTGGCA'3, Kai1: 5'CTGTGAGAAGACGAAGGA'3, 5'TGGAGAAGGCTCAGGCGTG'3, GAPDH: 5'CTCCCTCAAGATTGTCAGCA'3, 5'GAGTGGCAGTGATGGCAT'3, FST: 5'GTGAACGACAATACTCTCT'3, 5'GGTTTGTTCTTCTTGTTCA'3, Arfd1: 5'CTGCCTCCACTGTGAAAAGA'3, 5'CTTCTGTAAACTGCTGCT'3, TPT1: 5'CTTGAAGAACAGAAACCA'3, 5'TAGTCCAGTAGAGCAACC'3, Camk2d: 5'CCTCTACATCTTGCTGGT'3, 5'CTGTGTCCCATTCTGGTGA'3, CXCL1: 5'CTTCAAGAACATCCAGAGT'3, 5'CATTCTTGAGTGTGGCT'3, Eef1a: 5'CAGGCACATCTCAGGCT'3, 5'AGTGGCTTACACTTTGG'3.

### Intracerebral stereotactic injections

N32 tumor cells (2 × 10^3 ^cells) and embryonic neural progenitor cells ST14A (2.5 × 10^5 ^cells) were stereotactically co-injected into Fisher rats (as described in [7]) using 5 animals/group. Animals were anesthetized with Isoflourane (2% in NO and O_2_) and given Marcain Adrenalin (2.5 mg/ml+5 μg/ml, Astra Zeneca, Sweden) subcutaneously into the skin covering the scull, before stereotactically (Kopf Instruments, Tujunga, CA) given intracerebral inoculations into the right striatum (anterior, 1.7, mediolateral -2.5, ventral -4.5 measured from the bregma and the dural surface). Injection of cells was made using a Hamilton syringe in a maximum of 3 μl RPMI medium supplemented with 1% Fisher rat serum. All animals were treated according to the Swedish guidelines for humane treatment of laboratory animals and the experiments were approved by the Lund/Malmö animal ethical committee. The animals were monitored daily and examined for any signs of distress, ataxia, abnormal movements, appetite loss or decrease in activity and sacrificed upon showing motor function deficits according to the Swedish guidelines for humane treatment. Kaplan Meier Survival curves were analyzed using the chi-square test with a p-value of p = 0.0035 between the groups.

### Immunocytochemistry and Immunohistochemistry

Fluorescent immunohistochemical staining was performed by rinsing cell slides in KPBS, blocking in appropriate serum 1 h followed by primary antibody incubation; polyclonal rb anti-TPT1 (10824-1-AP Protein Tech Group, Chicago, IL), blocking goat anti-Follistatin (AF669 R&D systems, Germany), biotin conjugated hamster anti-CD81 monoclonal (TAPA1, 559518 BD Pharmingen), rb anti-AXL (4977 Cell Signaling Tec.) overnight in 5% NDS, 0.25% Triton X-100. This was followed by rinsing and incubation for 2 h with secondary antibodies donkey anti-goat Cy2, streptavidin Cy2, donkey or rabbit Cy2 (all 1:400, Jackson, West Grove, PA). The sections were mounted on a cover slip using PVA-DABCO. The usage of patient glioblastoma multiforme tumor samples has been accepted by the Local Ethical Board of the University of Lund. Tumor pieces from surgical resection were transferred to IMDM cell culturing medium (GIBCO-Invitrogen, Carlsbad, CA, USA) before being snap-frozen in Isopentane at a temperature of -50°C. Tissues were then kept in -80°C until sectioning on a cryostat. Seven μm thick sections were cut and mounted directly onto glass slides. For immunohistochemical staining, sections were fixed in room-tempered acetone for 10 minutes and rinsed in PBS (GIBCO-Invitrogen, Carlsbad, CA, USA). To permeabilize the cells, PBS containing 0.1% saponin (Riedel-de Haen, Seelze, Germany) was used as washing buffer and diluent in all steps. Sections were blocked with 5% goat serum (Jackson ImmunoResearch Laboratories Inc. West Grove, P.A, USA) for 20 minutes and incubated with the primary antibody mouse anti-human follistatin (R&D Systems, Minneapolis, MN, USA) for 60 minutes in room temperature (RT). Sections were then washed and incubated for 30 minutes in RT with the secondary antibody, goat-anti mouse Alexa 488 (Molecular Probes, Eugene, Oregon, USA). The slides were mounted wet using Pro-Long Gold anti-fading reagent, (Molecular Probes), with nuclear staining (DAPI). As negative control, the primary antibody was omitted. Stained cells were analyzed by the use of a light microscope (BX-60, Olympus America Inc., Melville NY, USA), equipped with a mercury lamp and filters for fluorescence (U-MWG, U-MWB, U-MWU, Olympus). Images were taken in 20× magnification using an Olympus Color View digital camera and captured using the analySIS^® ^Image analysis software (Olympus).

### Western Blot

Western blots were performed according to protocols from Bio-Rad Laboratories, CA. The protein analysis was performed using the primary antibodies; polyclonal rb anti-CXCL1 (1:1000 ab17561 Abcam, Cambridge, UK), goat anti-Gas6 (1:1000, AF986 R&D systems, Germany). Subsequent secondary antibody staining was done using: goat anti-rabbit IgG-HRP, donkey anti-goat IgG-HRP (sc-2004, sc-2020, Santa Cruz Biotechnology) 1:5000.

### *In vitro *proliferation assay

Tumor cells (N32: 600 cells/w, N25/N29: 1000 cells/w) were seeded into 96 well plates either alone or together with irradiated progenitor cells (10 000 rad; 37 Cs source) (HiB5/ST14A/RN33B: 10 000–20 000 cells/w). After allowing the cells to attach, antibodies against FST/CD81/CXCL1/AXL/GAS6/TPT1 (as described above) were added at concentrations suggested by the manufacturer (0.1–1 μg/ml). Recombinant follistatin protein was added at a concentration of 5–10 nM to the tumor cells. After culturing the cells at 37°C for three days they were pulsed with 0.5 μCi ^3^H-thymidine (Amersham, Buckinghamshire, UK) for 6 h and the cells were subsequently harvested in a Tomtec cell harvester and ^3^H-thymidine incorporation was measured using a Wallac 1450 Microbeta Liquid Scintillation Counter (Turku Finland). The assays were repeated three times with a minimum of quadruplicates per assay. Statistical analysis was measured using the GraphPad program (GraphPad Prism version 4.00 for windows, San Diego, CA). Differences between means of two groups were tested using a t-test/as well as Mann whitney test, with significance P < 0.05. These data are expressed as fold changes based on the effect of NPC on tumor growth alone.

## Results

**Figure 1:**
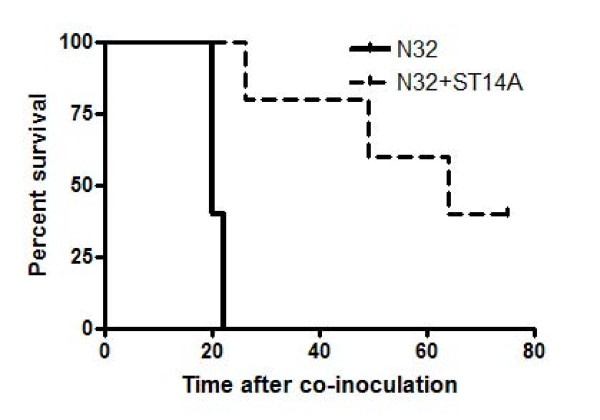
**Prolonged animal survival is seen after co-inoculation of glioma with NPC**. Co-inoculation of NPC together with glioma cells into the striatum of experimental fisher rats result in reduced tumor growth. The glioma N32 (2 × 10^3 ^cells) was stereotactically co-injected with the NPC, ST14A (2.5 × 10^4^) into the striatum of fisher rats (n = 5/group). Antitumor properties for the other NPC lines are shown in our previous publications (Staflin et al. 2004, 2007). The percent survival was followed over time and statistical analysis of the Kaplan Meier curve was done using a chi square test **p = 0.0035.

In our previous study, the two embryonic rat progenitor cell lines HiB5 and ST14A showed a strong antitumor effect on syngeneic malignant glioma models *in vivo *[7,8]. Previous successful treatment regimens were based on using tumor verses NPC cell ratios ranging from 1:5–1:100. To gain insight into the efficacy in treating tumors with NPC, new experiments were designed to maximize the antitumor effect using a 1:125 ratio. This was done in order to identify factors contributing to the antitumor effect. Injections of the NPC cell line ST14A into the striatum of Fisher rats show antitumorigenic properties towards the malignant glioma N32 if co-inoculated into the striatum. All animals receiving N32 tumor had to be sacrificed by day 22 post injection due to tumor related symptoms whereas animals co-inoculated with ST14A showed a prolonged lifespan and a cure of up to 40% of the animals (Figure 1). In cured animals there was no trace of tumor growth after six months as investigated by histological examination (data not shown).

### Classification of differently expressed genes in progenitor cells and glioma cells

**Figure 2:**
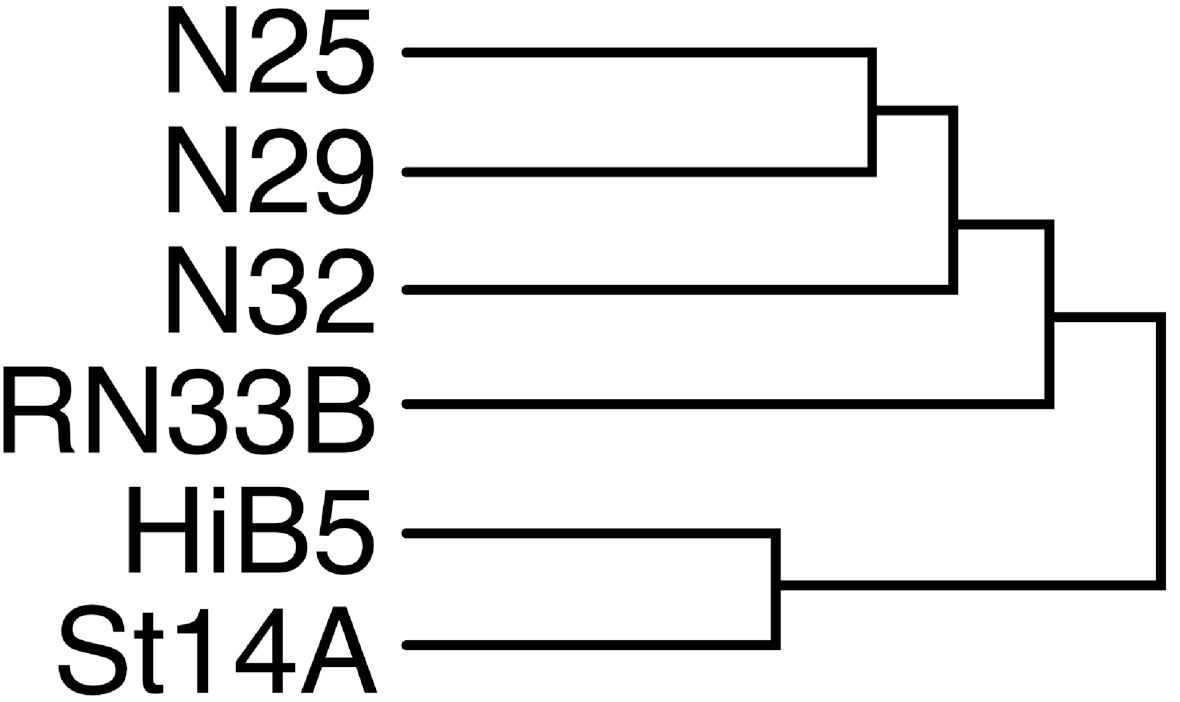
**NPC with antitumor properties show a high genetic semblance**. Dendrogram of the genetical semblance between the gliomas N25, N32 and N29 as well as their relationship with the NPC HiB5, ST14A and RN33B. The arrangement is based on the initial array after pre-processing and filtering, before further steps for selecting genes were performed. The two NPC with antitumor effect cluster together and RN33B lacking antitumor effect clusters together with the gliomas. Hierarchical clustering and analysis was done with the software package BASE 1.2.17 (available at http://base.onk.lu.se). For the whole cluster analysis see Additional file 1.

Microarray analysis was carried out to screen the tumor cells and NPC with the goal to find putative genes involved in glioma growth and invasion. Thereby, we hoped to identify factors that could be used for future therapy, circumventing the use of elaborate NPC transplants. Comparative analysis was carried out on the malignant gliomas N32 and N29, which have been shown to be sensitive to treatment with NPC, versus the glioma N25 that showed no response to treatment. The initial array clustering analysis revealed the level of genetic resemblance between the cell lines (Figure 2). The dendrogram, which is based on the complete array results after pre-processing and filtering, shows that the two NPCs (HiB5/St14A) which display antitumor properties cluster together. The cell line RN33B, which has no antitumor properties, show a greater resemblance with the tumors (the whole cluster analysis is shown in additional file 1). The gene expression profile of the gliomas were cross compared with the array data from the embryonic neural progenitor cells HiB5/ST14 and RN33B, where the latter has no antitumor properties. Additional cross comparisons within tumor- and NPC-groups were also made. The top regulated genes for each comparison are shown in Table 1. Genes were sorted into different groups of interest such as growth factors, metabolism, cell cycle, cell death and then screened in proteomic databases. We chose to continue with 10 genes for quantitative PCR analysis and 6 of those were also confirmed by protein expression analysis using either western blots or immunocytochemistry (Table 2). AXL was not present on the array chip, but is the receptor for Gas6 and was therefore added to the protein analysis. All gene products that were selected for characterization by protein expression levels were either secreted or membrane-bound proteins. Some have been shown previously to be of importance for tumor growth, invasion, metastasis and/or chemotaxis, such as Gas6/AXL and CXCL1 [26,27].

**Table 1:** The top regulated genes from the array for each comparison.

Gene	**Fold change **N29 and N32 vs N25
**Cryac**	33.06
**Prss1**	8.41
**Prrx1**	6.08
**Lgals3**	5.04
**Faf1**	4.45
**Kcnd2**	0.34
**Camk2n2**	0.32
**Tgfb1i4**	0.31
**Igfbp5**	0.24
**Fst**	0.14

**Gene**	**Fold change **ST14a and Hib5 vs Rn33b

**Fst**	155.25
**Cryac**	62.76
**Smgb**	41.11
**Prss1**	35.74
**Gas6**	11.90
**Hfb2**	0.26
**Dbi**	0.19
**Map17**	0.15
**Pitx2**	0.12
**Col18a1**	0.06

**Gene**	**Fold change **glioma/NPC

**Col3a1**	7.13
**Pga5**	2.75
**Nme1**	1.94
**Rasa1**	1.84
**Hfb2**	1.81
**Gas6**	0.12
**Mch**	0.10
**Mcl1**	0.09
**Smgb**	0.07
**Fst**	0.04

Microarray comparisons. Shown are the fold changes of the five most up- or downregulated genes for the following comparisons: N29 and N32 vs. N25, ST14A and Hib5 vs. RN33b, Glioma vs. NPCs. Statistical analysis was performed using the software SAM, which allows the detection of falsely positive genes (family-wise error rate) by averaging the amount of genes classified as significant over chosen permutations of data between the groups. The false discovery rate was set to false discovery rate ≤ 1 10^-4^.

### Validation of candidate genes

To confirm the gene expression of the selected genes on RNA and protein levels we performed RT-PCR, immunocytochemistry and western blot analysis (Table 2).

**Table 2:** Expression profiles of selected genes and proteins from microarray analysis, RT-PCR, immunocytochemistry and western blot analysis.

GENE CELL	N25	N32	N29	RN33B	ST14A	HIB5	ANALYSIS
Follistatin	+	-	+/-	-	+++	+++	**Microarray**
	+	-	-	-	+++	++	**RT-PCR**
	+	+/-	-	-	+++	+++	**Protein exp**

GAS6 (growth arrest specific protein 6)	+/-	+/-	+/-	+/-	+++	+++	**Microarray**
	+/-	+/-	+/-	-	+++	++	**RT-PCR**
	-	-	-	+/-	++	++	**Protein exp**

TAPA1/CD81	+	missing	++	+	+++	+++	**Microarray**
	+	+++	+	+/-	+++	++	**RT-PCR**
	++	+++	+++	-	++	+++	**Protein exp**

AXL	missing	missing	missing	missing	missing	missing	**Microarray**
	++	+	++	+	+++	++	**RT-PCR**
	+++	++	+++	-	++	+++	**Protein exp**

GRO1/CXCL1 (chemokine (C-X-C motif) ligand 1)	+++	++	++	++	+	+	**Microarray**
	++	+++	+	-	++	++	**RT-PCR**
	-	+++	++	-	-	-	**Protein exp**

TPT1 (tumor protein, translationally controlled 1)	++	+	++	+/-	+++	+++	**Microarray**
	+++	++	+	+/-	++	+	**RT-PCR**

TRIM23/Arfd1 (tripartite motif-containing 23)	+	+	+	+	+++	+++	**Microarray**
	+	+	+	++	+++	++	**RT-PCR**

CD82/Kai1	++	++	+++	+	+++	+++	**Microarray**
	+++	++	++	+	+++	+++	**RT-PCR**

Camk2 (Ca/calmodulin dependent protein kinase typeII)	++	+	+	+	+++	++	**Microarray**
	++	+++	+	+	++	++	**RT-PCR**

Eef1a (Elongation factor 1 alpha)	++	+	+	+	+++	+++	**Microarray**
	+++	+	+	+	++	++	**RT-PCR**

The order of the scores in the table is 1) microarray, 2) RT-PCR, 3) protein analysis. The selected and compared genes were: Growth arrest specific 6 (gas6), follistatin (fst), AXL receptor tyrosine kinase (missing on the array), translationally-controlled 1 (TPT1), Kai (CD82), CD81 (Tapa-1), ADP-ribosylation factor domain protein 1 (Arfd1), Calcium/calmodulin-dependent protein kinase II (Camk2), growth regulated protein precursor CXCL1 and Eukaryotic translation elongation factor 1 alpha 1 (Eefa1). The levels of expression are compared within each method of measurement used and expressed within each group as +++ for the strongest expression measured. ++ was set for values ranging from 45–70% of the highest score (+) for 15–44% of the highest score, (+/-) for 1–14% of the highest score and (-) for no expression detected.

**Figure 3:**
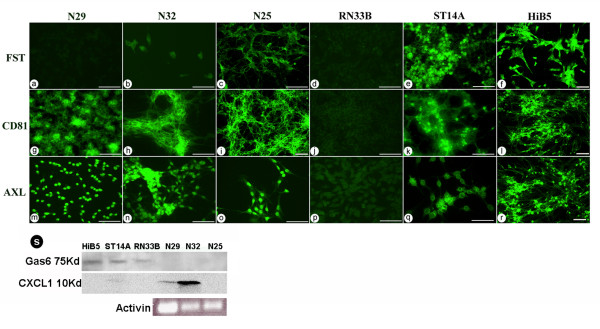
**Validation of protein expression**. Immunocytochemical analysis of protein expression of (a-f) Follistatin, (g-l) CD81, (m-r) AXL, Scale bars indicate 50 μm. (s) total protein expression analysis of the proteins Gas6 and CXCL1 comparing gliomas (N25, N29, N32) and NPC (HiB5, ST14A, RN33B). As well as RT-PCR analysis of activin expression in the tumors.

Genes highly expressed in the NPC displaying antitumor effects (HiB5/ST14A), according to RNA and protein levels (Figure 3, table 2), were follistatin, Growth arrest specific 6 (Gas6) and ADP-ribosylation factor domain 1 (Arfd1). Expressed genes found in both the tumor group and the NPC with antitumor effect were Camk2, CD81, Kai1, TPT1 and AXL. These genes were not expressed at all or less expressed by the control embryonic cell line RN33B which does not display any antitumor effects. Eef1a was differentially expressed in the different types of tumors and NPC.

Histology of protein expression revealed high follistatin expression by HiB5 and ST14A with very weak expression in N25 and no expression in the other cell lines, correlating with RT-PCR and microarray data (Figure 3a–f). Since follistatin is an activin inhibitor [18], we screened the tumors for activin expression using RT-PCR. All three gliomas expressed activin on the RNA level (Figure 3S). Gas6 is a secreted protein shown to be involved in tumor cell proliferation and invasion. Expression was found in HiB5 and ST14A, with lower levels in RN33B and no expression in the tumors (Figure 3S). The Gas6 receptor AXL and the cell surface molecule CD81 were abundantly expressed on the tumors and on HiB5/ST14A (Figure 3, g–r).

The chemokine/growth factor CXCL1 protein was exclusively expressed by the sensitive tumors N32 and N29 (Figure 3S). CXCL1 has previously been implicated in promoting angiogenesis and oncogenesis in various cancer types, including gliomas [19-21]. The RNA and protein expression levels correlate for the majority of the selected targets with the following exceptions: TPT1 (Tumor protein, translationally-controlled 1) which is a translationally controlled protein, CXCL1 and partially for Camk2.

### *In vitro *functional assays demonstrating effects on tumor proliferation

To get a preliminary evaluation of the selected genes in the interaction of glioma cells with NPCs, we performed a functional *in vitro *assay measuring cell proliferation. *In vivo *there is a slower progression in tumor growth with time in the presence of NPC [7,8]. This is mimicked by the inhibition of tumor proliferation if co-cultured with NPC *in vitro *[7]. In co-cultures of tumor cells together with irradiated non proliferating NPC we have in previous studies shown that HiB5 and ST14A can inhibit the tumor proliferation of N32 and N29 *in vitro*, but not of N25, thereby giving us a potential model system for measuring the antitumor effect seen *in vivo*. RN33B has no effect on tumor growth *in vivo *or *in vitro *[7]. We here performed functional assays using antibodies against the proteins follistatin, Gas6, AXL, CXCL1, CD81 and TPT1, added to either single tumor or progenitor cell cultures or co-cultures of tumor and progenitor cells at day 0.

Co-cultures of either HiB5 or ST14A with N32/N29 show an inhibition in tumor proliferation as compared to single cultures of tumor cells. Counts per minute (CPM) for single cultures with N32 ranged between 5796–13069 ± 180–200 cpm and for N29 between 1115–7133 ± 73–235 cpm depending on the number of cells used. The relative inhibition by HiB5 or ST14A decreased the proliferation of the tumor N29 by 77–91% and 76–94% respectively. For the glioma N32, the proliferation decreased with 89–96% and 57–88% in the case of treatment with HiB5 or ST14A respectively. N25 was insensitive to the antitumor effect elicited by the progenitor cells (data not shown). This level of inhibition on tumor cell division by the NPC was set as the baseline level (1), without the addition of antibodies. Co-cultures were subsequently treated with antibodies at day0 and measurements were made at day 3. Addition of antibodies at day 0 either increased the inhibition of NPC on glioma proliferation (values below 1) or counteracted the inhibition of cell proliferation (values above 1). Some of these antibodies are not confirmed as blocking antibodies and hence there is a possibility that they do not target the functionality of the protein in an optimal way. On the other hand, controls using isotype IgG did not show any effect (data not shown), suggesting that the specific antibodies interacted with target protein functionality.

**Figure 4:**
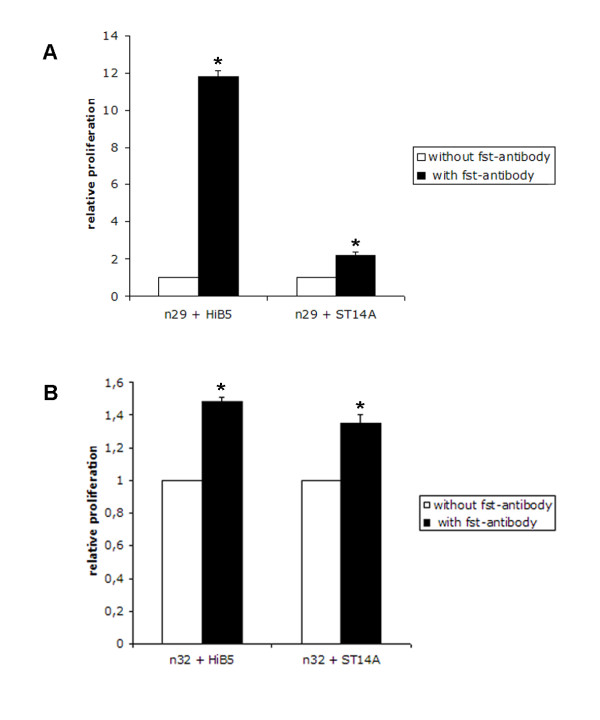
**Blocking antibody against the follistatin protein results in an increased tumor proliferation**. Co-cultures of tumor N29 (A) and N32 (B) together with irradiated NPC (HiB5/ST14A), were treated by adding an antibody targeting the follistatin protein. Cells were seeded together and allowed to attach before addition of the antibodies. The plates were pulsed with ^3^H-thymidine after 3 days in culture and cell division was measured as the amount of incorporated ^3^H-thymidine. White bars show the proliferation of NPC + tumor control cultures and black bars show the proliferation after the addition of follistatin antibody. Mean ± stdev: Statistical analysis was made using t-test and Mann Whitney Wilcoxon rank sum test * P < 0.05.

**Figure 5:**
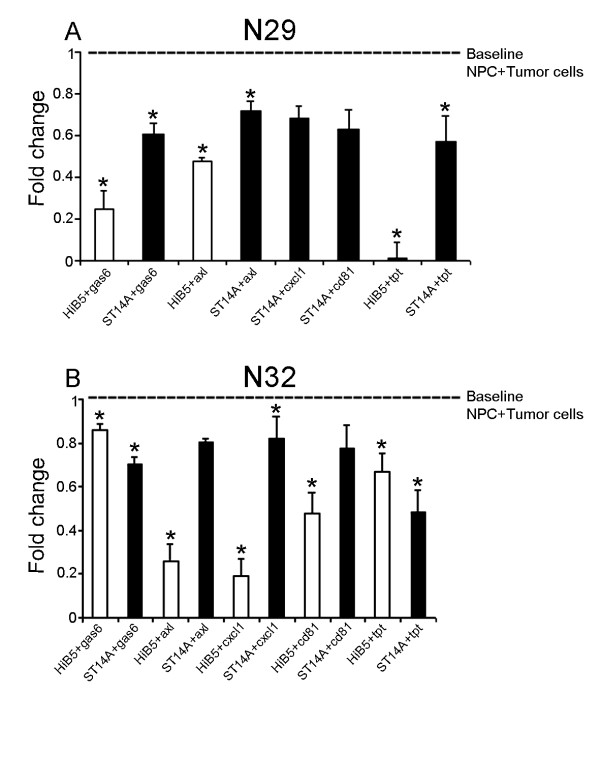
**A decrease in tumor proliferation after the addition of antibodies against the genes Gas6, AXL, CXCL1, CD81 and TPT1 was observed**. Co-cultures of tumor N29 (A) and N32 (B) together with irradiated NPC (HiB5/ST14A), were treated by adding antibodies against Gas6, AXL, TPT1, CXCL1 and CD81. Cells were seeded together and allowed to attach before addition of the antibodies. The plates were pulsed with ^3^H-thymidine after 3 days in culture and cell division was measured as the amount of incorporated ^3^H-thymidine. The result is expressed as fold change, where 1 represents the inhibition by NPC alone on the tumor (dashed line). Values below 1 show a further decrease in tumor proliferation due to the addition of antibodies. White bars show HiB5 and black bars show ST14A cultures. Mean ± stdev: Statistical analysis was made using t-test and Mann Whitney Wilcoxon rank sum test * P < 0.05

Follistatin protein was shown to be expressed exclusively by HiB5/ST14A (Figure 3f–g). The addition of a blocking antibody to follistatin in co-cultures increased the tumor proliferation, indicating that follistatin is involved in the inhibition of tumor proliferation. The antibody significantly counteracted the HiB5 cells inhibitory effect on glioma proliferation and to a lesser extent influenced the effect of ST14A on tumor growth (Figure 4). Addition of blocking antibody to single cultures of tumor (N32/N29/N25) had no influence on tumor growth (data not shown). Addition of follistatin antibody to cultures of NPC weakly decreased their proliferation, indicating that follistatin might have a paracrine/autocrine regulatory function in cell division for the NPC themselves (data not shown). Further, the addition of recombinant follistatin protein to cultures of N29 showed an inhibition of proliferation by 33% compared to the control after three days. Control cultures (N29) had a proliferation of; CPM 2699 ± 95 and the addition of follistatin resulted in a decreased CPM to 1818 ± 140. Addition of antibodies against the other five selected genes to co-cultures either had no effect or resulted in a reduction of tumor proliferation (Figure 5A–B). This suggests that some of these proteins may be involved in promoting tumor growth. This can be exemplified by the Gas6/AXL system where the addition of antibodies against the protein Gas 6 potentiated the inhibition elicited by the NPC on the N29 tumor and to a lesser extent on N32 (Figure 5A–B). This was further strengthened by the fact that addition of antibodies against the Gas6 receptor AXL also increased the inhibition of tumor proliferation on N29 and N32 using HiB5 (Figure 5A–B).

Addition of antibodies against CXCL1, CD81, and TPT1 had various effects either inhibiting the tumor proliferation or showing no significant changes upon the tumors (Figure 5).

### Follistatin expression by human gliomas

**Figure 6:**
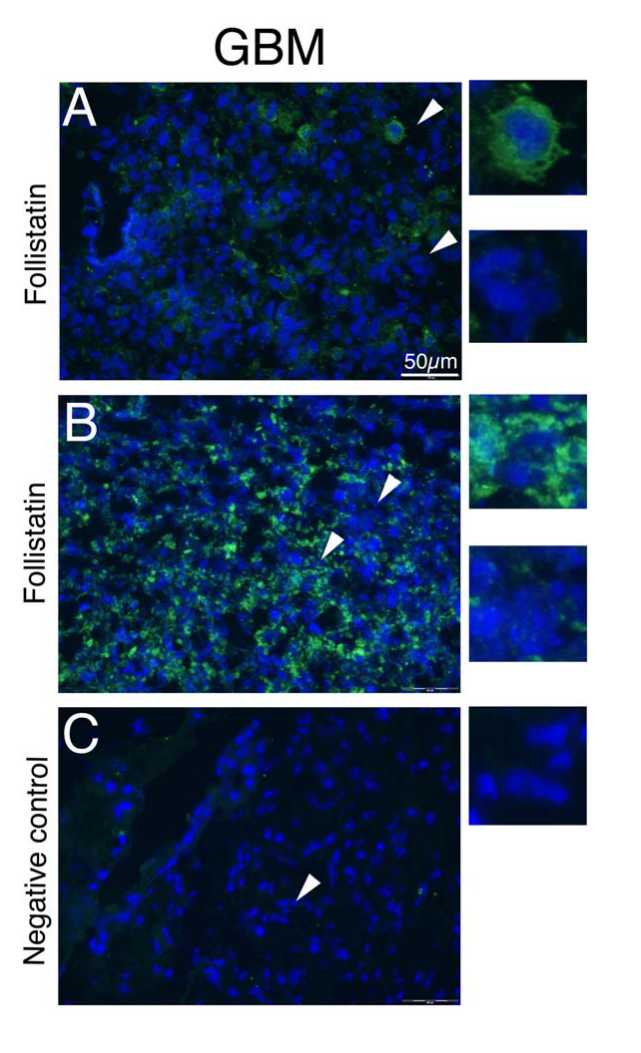
**A subpopulation of grade IV human GBM display follistatin expression**. Figure 6. Follistatin expression by subsets of tumor cells within the glioblastoma multiforme (GBM) tumor cell bulk. Samples from GBM patients were freeze sectioned and subsequently stained using a follistatin antibody against the human antigen. A) Central tumor sections of patient A. A few selectively follistatin positive cells are seen within the central portions of the tumor. B) Central section from patient B. Follistatin expression is seen in a subset of tumor cells within the central bulk of the tumor. The histological examination of patient A and B tumors displays a heterogeneity within the tumor bulk with both positive and negative tumor cells. As negative control, the primary antibody was omitted, C. Scale bars indicate 50 μm.

To validate the expression of follistatin in human gliomas we stained four human glioblastoma multiforme (GBM) grade IV tumors with an antibody recognizing follistatin. Tumor stroma as well as some infiltrating microglia/macrophage-like cells were found to be follistatin positive. In addition, there were distinct sub-populations of tumor cells within the tumor bulk, which were follistatin positive (Figure 6A–B). Two out of four GBMs were low to moderately positive (A) and two showed a higher degree of positivity (B). Two representative tumors are shown in figure 6. This suggests that follistatin has a role in glioma growth and might be of interest as a potential marker for glioma grade as well as for tumor progression.

## Discussion

The majority of the glioblastoma cases are primary glioblastomas, hence developed de novo displaying aberrations in the EGFR expression, p16^INK4a^, and PTEN mutations [22,23]. Secondary glioblastomas arise from the progression of lower grade tumors and often display TP53 mutations. The characterisation of relevant protein markers and targets for glioma are identified with the ATLAS project and confirms the relevance of genes such as CD81, and InhibinB [24]. The underlying genetical aberrations in our glioma models are currently unknown but most gliomas have one or more dysfunction in signalling pathways regulating growth factor signalling (EGF/PDGF), p53/Rb tumor suppressors and or PI3K/PTEN pathways [23,24]. The TGFβ-family also plays a role in the progression of gliomas and consists of activins, inhibins and bone morphogenic proteins (BMP). This family regulates known oncogenes such as c-myc as well as p15^INK ^and P21 ^CIP ^and have also been found to be responsible for the dysregulation of growth factor pathways in glioma [25]. Our data implies that the TGFβ family member follistatin is of importance for regulating glioma growth. The relevance and importance of the selected genes presented in our work is shown by the fact that the majority are found to be over expressed in human glioblastoma multiforme. These include CXCL1, AXL and Gas6 which provide negative prognostic markers for the disease [26,27].

The aim of this study was to find candidate genes involved in affecting tumor proliferation and progression based on previous findings that specific progenitor cells are able to affect glioma growth in the CNS [7,8,28-30]. By characterizing the two embryonic NPCs for their properties in targeting tumors we could potentially isolate and supply a tool for future treatments of highly malignant gliomas without using cell based therapy. Further, the characterization of the tumors together with the NPC would reveal genes and proteins important for tumor growth and invasion that might be transferred to human tumors. The initial microarray analysis revealed that in fact the two NPC with antitumor properties cluster together. This shows a certain similarity on the gene level between these lines translating into their ability to affect tumor growth. The genes that were selected from the microarray analysis, based on known localization and function, were confirmed on RNA and protein levels. Highly expressed genes in HiB5 and ST14A such as follistatin, a TGFβ-family antagonist, and Gas6 are known to be involved in cell proliferation and migration. In addition they have the ability to influence the growth of tumor cells. NPC have a great self renewing and proliferative potential [31] and might rely in part on similar signaling pathways as tumor cells originating from the brain, such as gliomas. This would explain the found similarity between the cell populations as well as their ability to influence each other as shown in previous work [7,8]. One example is the importance of the TGFβ family and SMAD signaling in regulating stem cell multipotency, self renewal and differentiation as well as their role in regulating tumorigenesis [32].

*In vivo *we observe a decreased tumor growth rate in the presence of NPC. *In vitro *models are inefficient in mimicking the complexity of the *in vivo *environment, but can serve the purpose of illustrating important mechanisms that might be a part of the *in vivo *scenario, such as inhibition of tumor proliferation. The *in vitro *screening assays performed in this study, using specific antibodies against protein candidates, validated the tumor-inhibiting or promoting effect of those proteins and gave valuable hints that underline the importance of the mechanisms behind those effects. Blocking follistatin secretion from HiB5/ST14A in tumor co-cultures resulted in an increased proliferation rate of the tumor cells. This, together with the finding that the addition of recombinant follistatin on tumor cells inhibited N29 proliferation by 30%, might suggest a potential role for follistatin in regulating glioma growth. Follistatins are extracellular proteins, which bind TGFβ-family members such as BMPs and activins (especially activin A) with high affinity, blocking their receptor interaction [18]. The TGFβ-family members are potent regulators of cell growth, cell survival and differentiation in many different cell types [25,33]. In malignant disease, family members such as TGFβ, BMP and activin can act as tumor suppressors or as promoters of tumor growth [32-36]. The activin and follistatin balance has been shown to be dysregulated in numerous solid tumors, such as prostate cancer and hepatocarcinogenesis [37,38] and the development of resistance to TGFβ growth inhibition is a key event in tumor progression [37,25]. We show that rat gliomas express activin, however there are other TGFβ-family members that might be relevant to look at as potential targets for follistatin regulation, such as the BMPs, which have similar growth and survival regulatory functions. Most cell types and some tumors respond with growth arrest to TGFβ-family members such as activin, by many different mechanisms, including upregulation of cell cycle inhibitors. This is usually mediated via SMAD dependent signaling pathways [39]. In other instances, the tumor cells have become non-responsive to cell cycle regulation by TGFβ-family members and instead produce TGFβ/activin/BMP that have a beneficial role for the tumor development e.g. modulating tumor matrix, adhesion molecules and suppress the immune system [18,40-45]. This has been shown to occur in part via SMAD independent signaling pathways [18,39]. The production of follistatin by NPCs resulting in tumor-growth inhibition could be mediated by many different mechanisms *in vivo *e.g. inhibition of immuno-suppression, dysregulation of matrix production or direct anti-proliferative effects on the tumor. The increase in growth after the addition of follistatin antibodies *in vitro*, or the direct inhibitory effects of recombinant follistatin on glioma proliferation might hence influence important cell maintenance signals or directly impact cell proliferation e.g. by modulating the matrix. We evaluated the expression of follistatin in several grade IV human gliomas and found a subpopulation within these tumors which express follistatin. This might suggest that the tumor acquires similar mechanisms to increase the resistance to growth inhibitory effects, mediated by follistatins, as seen with other TGFβ-family members such as Activin and TFGβ. With higher grade, gliomas may become more resistant to growth inhibitory signals from TGFβ-family members since it confers a survival advantage for the tumor. In addition, becoming resistant to these growth inhibitory signals is vital to escape the effects of infiltrating immune cells secreting inhibitory BMPs and TGFβ. In support of this notion is the fact that the follistatin homolog follistatin like protein 1 has been shown to be a negative prognostic factor and is abundantly expressed in high grade gliomas [46]. It would be of interest to investigate if the expression of follistatin increases with glioma grade in accordance with follistatin like protein [46]. Our rat glioma cell lines cannot be graded according to the WHO but have similarities to grade III-IV gliomas. Human gliomas are of much higher cellular diversity than the rat glioma cell lines used in this study. Therefore, only a fraction of human tumor cells within a glioma may respond in the same way as in our experimental model. This also demonstrates that the heterogeneity of the cells within a tumor make them potentially respond different to treatment regimens and might explain why some of our experimental tumors do not respond to NPC treatment. The glioma N25 is one such example, where the tumor might have gained a resistance to TGFβ family related growth inhibitory effects. This cell line was shown to be weakly positive for follistatin in culture in contrast to the gliomas which respond with growth arrest to the NPC.

Follistatin is a good candidate for the tumor inhibition observed by the embryonic progenitor cell lines HiB5 and ST14A. It will be of great importance to test the feasibility of the approach *in vivo*, to use tools for blocking the actions of follistatin in animals with gliomas and in animals receiving NPC treatments (using e.g. siRNA or antibodies). Indeed, our initial investigations of follistatin expression by the NPCs after transplantation to the rat brain suggest that they sustain the follistatin expression *in vivo *(K. Staflin, unpublished observation). These findings provide a basis for further investigation into the role of follistatin in cancer development *in vivo*.

In addition, several other proteins were identified which might be of importance for regulating the ability of the gliomas to grow and invade. The Gas6-AXL pathway is involved in tumor stimulating actions such as stimulating growth and invasion as well as in anti tumor actions, increasing leukocyte transmigration from vessels important in tumor targeting [47-51]. The Gas6/AXL pathway has also been shown to protect epithelial/endothelial cells from apoptosis and has been suggested to play an important role in tumor vascularization [27]. This implies that targeting the Gas6/AXL pathway in gliomas might not only have direct effects on tumor proliferation signals but could have secondary effects by decreasing the vascularization of the tumor and may present a good target for glioma therapy.

Our results show an increased inhibition of tumor growth by the NPC with the addition of either antibodies to these proteins, which point towards an implication of the importance for Gas6-AXL signaling in promoting tumor growth *in vitro *in the syngeneic rat glioma models. These results have relevance for the treatment of human GBM since both these proteins have been shown to be overexpressed in human gliomas and act as negative prognostic factors for the disease [27]. The N32 glioma was less responsive to the blocking of Gas6 and AXL signaling. This unresponsiveness might explain the difference in invasion capability of the tumors, with N29 being strongly invasive and N32 being less invasive [7]. Previous studies show that CXCL1 mediates glial progenitor cell proliferation during development of the brain but it has also been shown to have a role in the promotion of angiogenesis, oncogenesis in prostate cancer, in melanomas and in increased tumorigenicity of gliomas [19-21]. When antibodies specific for CXCL1, TPT1 or CD81 were added to tumor co-cultures with NPC, the suppressive effect by the NPC was significantly enhanced in several combinations of NPC and tumor, although to a varying extent. This clearly argues for a promoting effect of some of those factors on tumor growth. For CXCL1 and TPT1, this tumor proliferative effect is already known [21,26,52]. However, we were able to show that this proliferative effect also applies to the specific gliomas N32 and N29. The fact that addition of an antibody enhances the suppressive effect of HiB5 and ST14A on tumor growth gives hints that the suppressive effect of these NPC is not due to their secretion of TPT1, CD81 and CXCL1, Gas6/Axl but to other factors. Furthermore, identification of these proliferation promoting factors would allow the genetic modification of the NPC lines HiB5 and ST14A to optimize their effects for future experiments. CD81 has not yet been clearly identified as either tumor growth suppressor or enhancer. We were able to show that CD81 has a proliferative effect on tumor growth of N29 and N32. This is in line with findings in Geisert et al. 2002 [53] and argues for future experiments looking for options to suppress CD81 expression as a possible candidate for tumor treatment. Candidates, displaying both positive and negative effects on glioma proliferation were characterized. Specific candidates like follistatin and the Gas6/AXL system, point towards future experiments evaluating them in more detail as targets for future therapy of gliomas.

## Conclusion

Taken together, we have identified several putative factors that influence rat glioma growth and were able to further characterize the NPC cell line backgrounds from which these factors were secreted in our experimental setup. The factors identified might be useful starting points for performing future experiments directed towards a possible therapy against malignant gliomas.

## Competing interests

The authors declare that they have no competing interests.

## Authors' contributions

KS carried out the micro-array together with GH. KS carried out quantitative RT-PCR, Western blots and immunocytochemistry. TZ a nalyzed the micro-array data together with KS and took part in designing the validation studies. AD carried out the immunohisotchemistry on the human samples. CL participated in the design and coordination of the study and helped to draft the manuscript. All authors read and approved the final manuscript.

## Pre-publication history

The pre-publication history for this paper can be accessed here:

http://www.biomedcentral.com/1471-2407/9/206/prepub

## Supplementary Material

Additional file 1 **Figure S1**. Cluster analysis of micro array. Click here for file
